# Associations Among Preoperative Intrusive Thoughts, Postoperative Pain Intensity, and Thirst Within the Biopsychosocial Model: An Observational Study

**DOI:** 10.3390/healthcare14203480

**Published:** 2026-10-16

**Authors:** Hatice Özsoy, Muaz Gülşen, Askeri Çankaya, Ahmet Ragip İyiol

**Affiliations:** 1 Medical Laboratory Techniques Program, Burdur Vocational School of Health Services, Burdur Mehmet Akif Ersoy University, Burdur 15100, Türkiye; 2 Department of Surgical Nursing, Faculty of Health Sciences, Çukurova University, Adana 012500, Türkiye; 3 Department of Surgical Nursing, Faculty of Health Sciences, Fırat University, Elazığ 23270, Türkiye; 4 Department of General Surgery, Burdur State Hospital, Burdur 15100, Türkiye

**Keywords:** biopsychosocial model, intrusive thoughts inventory, pain, thirst

## Abstract

**Background/Objectives:** This study aimed to examine the associations of preoperative intrusive thoughts with postoperative pain intensity and thirst discomfort within a biopsychosocial framework. As a secondary exploratory objective, the study sought to estimate direct and indirect statistical associations among these variables using a path model. **Methods:** This observational, descriptive-correlational study included 130 patients who underwent laparoscopic cholecystectomy at the General Surgery Clinic of a state hospital in the Mediterranean Region between 1 August 2025 and 31 January 2026. Data were collected using the Patient Demographic Form, the Surgical Period Data Form, the Preoperative Intrusive Thoughts Inventory (PITI), the Numerical Rating Scale (NRS), and the Perioperative Thirst Discomfort Scale (PTDS). **Results:** The mean PITI total score was 18.78 ± 11.43, the mean NRS score was 5.23 ± 2.08, and the mean PTDS total score was 5.58 ± 2.72. Moderate positive correlations were found between PITI and NRS scores (r = 0.320, *p* < 0.001) and between PITI and PTDS scores (r = 0.303, *p* < 0.001). A weak positive correlation was also found between NRS and PTDS scores (r = 0.191, *p* < 0.05). In the secondary exploratory path analysis, the indirect statistical effect in the specified model involving postoperative NRS scores, preoperative PITI scores, and postoperative PTDS scores was statistically significant (indirect effect = 0.105, SE = 0.0448, 95% CI [0.0169, 0.192], *p* = 0.019), whereas neither the direct effect nor the total effect reached statistical significance. **Conclusions:** Preoperative intrusive thoughts were associated with postoperative pain intensity and thirst discomfort. These findings suggest that preoperative intrusive thoughts may be relevant to understanding postoperative symptom experiences within a biopsychosocial framework. Given the timing of the measurements, the exploratory indirect association should be considered hypothesis-generating rather than evidence of causal mediation.

## 1. Introduction

The biopsychosocial model considers illness and symptom experiences in relation to biological processes, psychological characteristics, and the individual’s social and clinical context [1,2]. It provides a conceptual framework for examining postoperative symptoms alongside individual perceptions, cognitive processes, and emotional responses [3,4]. In this study, the model guided the examination of associations among preoperative intrusive thoughts, postoperative pain intensity, and thirst discomfort.

Pain is a sensory and emotional experience associated with, or resembling that associated with, actual or potential tissue damage [5]. Postoperative pain is frequently encountered following surgery [6] and may persist after discharge [7]. Psychological factors have been discussed as possible contributors to differences in postoperative pain intensity among patients undergoing similar surgical procedures [8]. Examining preoperative psychological characteristics may therefore help describe the individual differences observed in postoperative pain experiences.

Thirst is an unpleasant subjective experience related to the regulation of hydration [9] and is frequently reported after surgery [10,11]. In addition to dryness of the mouth, lips, and throat, patients may experience anxiety, stress, and restlessness associated with thirst [10,12,13]. Some patients describe perioperative thirst as more distressing than pain or fasting [9], highlighting its importance in postoperative symptom assessment [14]. Preoperative fasting, fluid restrictions, anesthesia, and surgical stress have been identified as factors associated with postoperative thirst [11,15,16]. Surgical stress may also alter fluid regulation through antidiuretic hormone secretion [17]. These physiological and perioperative factors can be considered alongside individual differences in symptom perception and reporting within a biopsychosocial framework [18].

Preoperative intrusive thoughts have been associated with fear, anxiety, and depression, which may adversely affect patients’ quality of life [19,20]. Anxiety and depression have also been associated with different trajectories of perioperative thirst discomfort, with the direction of these associations varying [21]. Patients have described thirst as an experience accompanied by anxiety, desperation, and powerlessness [22]. More specifically, preoperative intrusive thoughts have been positively associated with fear of pain [23]. In this study, the term “intrusive thoughts” refers to the preoperative thoughts assessed by the Preoperative Intrusive Thoughts Inventory (PITI), including concerns about loss of control and the post-procedure period. Although these concerns overlap with aspects of preoperative anxiety, PITI scores should not be interpreted as equivalent to measures of general anxiety, depression, or intrusive thoughts in psychiatric disorders. The existing literature provides a rationale for examining the associations of these PITI-assessed thoughts with postoperative pain intensity and thirst discomfort.

Previous studies have examined postoperative pain and thirst in relation to patient comfort [24] and evaluated interventions addressing these symptoms [25]. Examining their associations with preoperative intrusive thoughts together within a biopsychosocial framework remains an area for investigation. Therefore, this study aimed to examine the associations of preoperative intrusive thoughts with postoperative pain intensity and thirst discomfort. As a secondary exploratory objective, direct and indirect statistical associations among these variables were evaluated using a path model.

The research questions are as follows:

- What are the levels of preoperative intrusive thoughts, postoperative pain intensity, and postoperative thirst discomfort among patients?
- Are patients’ demographic and clinical characteristics associated with preoperative intrusive thoughts, postoperative pain intensity, and postoperative thirst discomfort?
- Is postoperative pain intensity associated with postoperative thirst discomfort?
- What direct and indirect statistical associations among preoperative intrusive thoughts, postoperative pain intensity, and postoperative thirst discomfort are observed in the secondary exploratory path analysis?

## 2. Materials and Methods

### 2.1. Research Design

This observational, descriptive-correlational study, with assessments conducted during the preoperative and postoperative periods, was designed to examine the associations among preoperative intrusive thoughts, postoperative pain intensity, and thirst discomfort within a biopsychosocial framework. Direct and indirect statistical associations among these variables were also examined using a secondary exploratory path analysis. The study was not designed to establish a temporal or causal pathway among the variables. The study was reported in accordance with the STROBE guidelines [26].

### 2.2. Study Population and Sample

The study population consisted of patients who underwent laparoscopic cholecystectomy performed by a single surgeon at a public hospital in the Mediterranean Region of Türkiye between 1 August 2025 and 31 January 2026. The sample size was determined using the formula for a known population [27]. According to hospital records, 192 patients underwent cholecystectomy performed by the same surgeon in 2024, which represented the most recent completed annual surgical volume available at the planning stage and was therefore used as the reference population for sample size estimation. Using a 95% confidence level, a 5% margin of error, and *p* = 0.50 and q = 0.50, the minimum required sample size was calculated as 128 patients. The study was completed with 130 participants. This calculation was used to determine the minimum recruitment target and was not an a priori power analysis specifically designed for the correlation or exploratory path analyses.

**Figure 1:**
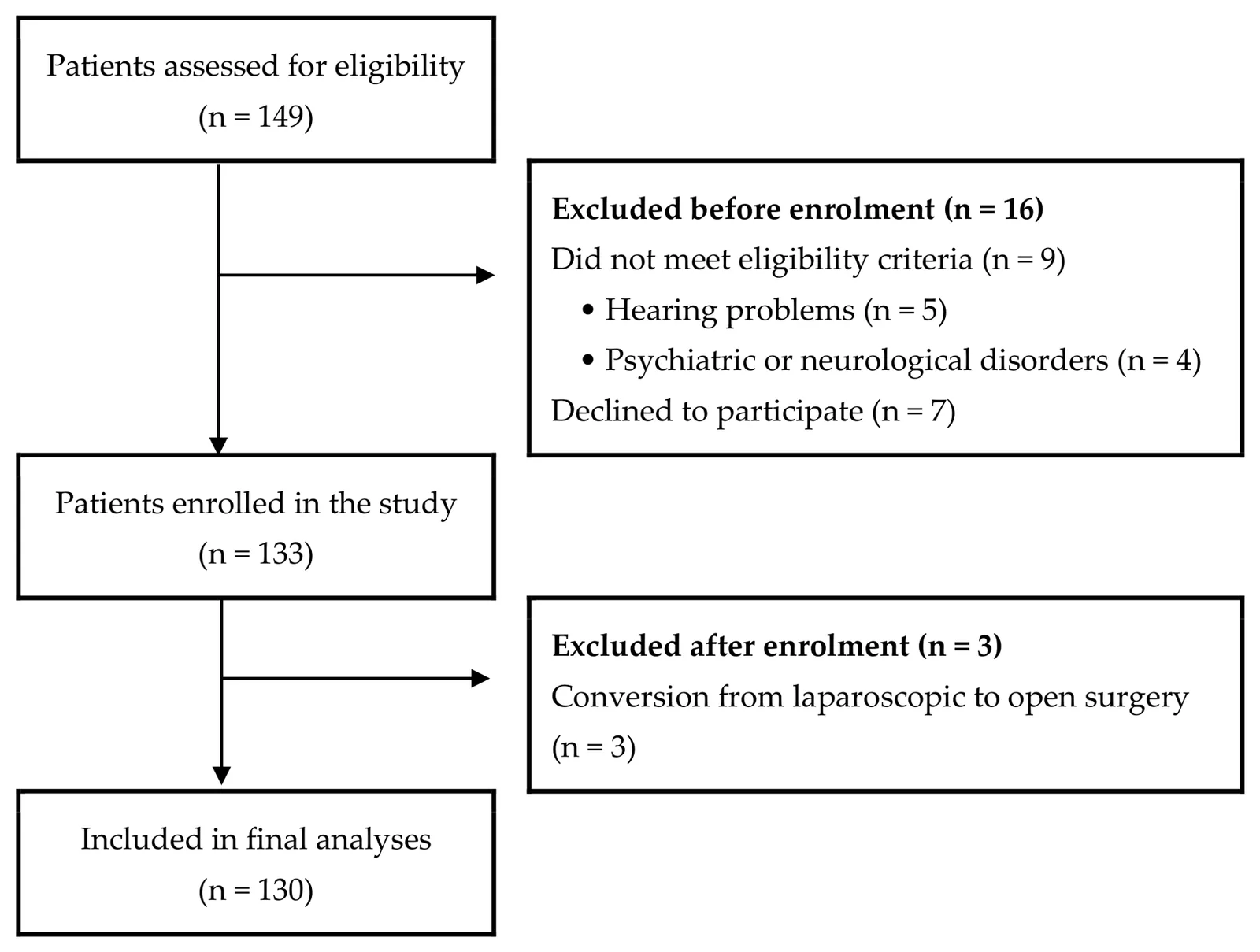
Participant flow diagram.

The study included patients who voluntarily agreed to participate, were aged 18 years or older, were classified as ASA I–III, underwent laparoscopic cholecystectomy performed by the same surgeon, and had no psychiatric or neurological disorders. Patients who declined participation, had communication difficulties, including hearing impairment that prevented effective communication during data collection, had psychiatric or neurological disorders, or required conversion from laparoscopic to open surgery were excluded. Psychiatric or neurological disease status was determined during the preoperative assessment based on patient reports checked against medical records; no structured psychiatric or neurological diagnostic interview was performed. During the recruitment period, 149 patients were assessed for eligibility. Of these, 9 did not meet the eligibility criteria (hearing problems, n = 5; psychiatric or neurological disorders, n = 4), and 7 declined participation. A total of 133 patients were enrolled, of whom 3 were subsequently excluded because of conversion to open surgery. Consequently, 130 participants were included in the final analyses (Figure 1).

### 2.3. Study Setting

The study was conducted between 1 August 2025 and 31 January 2026, at the General Surgery Clinic of a public hospital in the Mediterranean Region. Only patients undergoing elective laparoscopic cholecystectomy performed by the same general surgeon were included.

Patients were managed according to the institution’s standard perioperative care protocol. Oral intake was withheld from midnight on the night before surgery. Prophylactic antibiotics were administered during surgical preparation, and intravenous fluid therapy was initiated preoperatively.

A standardized postoperative analgesia protocol was followed for all participants. In the recovery room, patients received 1 g of intravenous paracetamol and 100 mg of intravenous tramadol hydrochloride. Following admission to the surgical ward, 20 mg of tenoxicam was administered, with a second dose given 12 h after the first. Routine treatment also included 40 mg of pantoprazole once daily, with the first dose administered upon admission to the ward. Postoperative intravenous fluid therapy on the ward consisted of 1000 mL of isotonic solution.

Including patients undergoing the same surgical procedure performed by the same surgeon and managed under a common perioperative care protocol was intended to reduce variability related to the surgical procedure, postoperative analgesia, fasting, and intravenous fluid therapy. However, detailed patient-level information on intraoperative anesthetic agents and doses was unavailable in the study dataset and could not be included in the analyses. Standardization of care should therefore not be interpreted as eliminating potential residual confounding related to anesthesia or other perioperative factors.

### 2.4. Data Collection Tools

Research data were collected using the Patient Demographic Form, Surgical Period Data Form, Preoperative Intrusive Thoughts Inventory, Numerical Rating Scale, and Perioperative Thirst Discomfort Scale.

#### 2.4.1. Patient Demographic Form

This form, developed by the researchers through a literature review [28,29,30], consists of a total of ten questions covering age, gender, educational status, weight, height, Body Mass Index (BMI), chronic disease status, smoking and alcohol use, and prior surgical experience.

#### 2.4.2. Surgical Period Data Form

This form, developed by the researchers based on a review of the literature [13,28,30], consists of three questions concerning the type of surgical procedure, the American Society of Anesthesiologists (ASA) physical status classification, and the duration of preoperative fasting for solid foods and liquids.

#### 2.4.3. Preoperative Intrusive Thoughts Inventory (PITI)

The PITI was developed by Crockett et al. (2007) to assess preoperative anxiety and its content [31]. Its Turkish validity and reliability study was conducted by Çakır and Karabulut (2019) [32]. The Turkish version consists of two subscales: anxiety about loss of control (items 1, 5, 6, 7, 11, 12, 15, 17, 19, and 20) and anxiety related to the post-procedure period (items 2, 3, 4, 8, 9, 10, 13, 14, 16, and 18).

In the present study, “intrusive thoughts” refers specifically to the preoperative anxiety-related cognitions assessed by the PITI. This construct should not be equated with generalized anxiety, negative affect, psychological distress, catastrophizing, or intrusive-thought phenomena described in psychiatric disorders.

The Likert-scale format for each question is as follows: (0) never, (1) sometimes, (2) often, and (3) most of the time. Scores on the scale range from 0 to 60. The interpretation of the scores is that as the score increases, the level of anxiety also increases [32]. In the study, the Cronbach’s alpha coefficient for the “anxiety about loss of control” subscale and the “anxiety related to the post-procedure period” subscale of the scale was determined to be 0.89, while that for the entire scale was 0.94.

#### 2.4.4. Numerical Rating Scale (NRS)

This scale is the most commonly used numerical measure for determining pain intensity and includes numerical values ranging from 0 to 10. Participants select one of these numbers to express the pain they are experiencing. The lowest value on the scale indicates the absence of pain, while the highest value indicates extreme pain [33].

#### 2.4.5. Perioperative Thirst Discomfort Scale (PTDS)

Developed by Martins et al. in 2017 [34], it was adapted into Turkish by Özsoy et al. (2023) [35], who also conducted a validity and reliability study. The scale consists of a total of six items and is evaluated using a three-point Likert-type rating scale. The ratings are: “Did not bother me” (0), “Bothered me a little” (1), and “Bothered me a lot” (2). The scale does not include any subscales; higher scores indicate increasing levels of thirst discomfort. The total possible score range for the PTDS is 0 to 12. The scale’s internal consistency coefficient (Cronbach’s alpha) was determined to be 0.87, indicating that the scale is highly reliable.

### 2.5. Research Data Collection

The study data were collected at the general surgery clinic between 1 August 2025 and 31 January 2026. Patients included in the study were visited at the clinic during the preoperative period and informed about the study. After the study’s purpose, scope, and data collection process were explained in detail, verbal and written informed consent was obtained from patients who agreed to participate.

The data collection process was conducted in two phases. The first phase covered the preoperative period. During this phase, patients were assessed at the clinic before being transferred to the operating room. The “Patient Demographic Form” was administered to determine the participants’ sociodemographic and clinical characteristics. Subsequently, the “Preoperative Intrusive Thoughts Inventory” was administered to determine the level of intrusive thoughts experienced during the preoperative period. Data recorded on the Patient Demographic Form and Surgical Period Data Form were obtained from patient self-reports and medical records. The second phase of data collection was conducted during the postoperative period. Postoperative pain intensity was assessed using the Numerical Rating Scale, and thirst discomfort was assessed using the Perioperative Thirst Discomfort Scale.

Postoperative pain intensity and thirst discomfort were assessed in the general surgery ward at the sixth postoperative hour, before oral intake was initiated. This time point was selected to assess both symptoms during the early postoperative period while patients were still fasting. Preoperative PITI scores were considered an antecedent cognitive/psychological characteristic potentially associated with both postoperative symptoms. Because PITI was assessed before pain intensity and thirst discomfort, and the two postoperative symptoms were assessed concurrently, the measurement schedule did not support the temporal ordering required to interpret PITI as a mediator of the relationship between postoperative pain intensity and thirst discomfort.

### 2.6. Data Analysis

Data were analyzed using IBM SPSS Statistics 20.0 (IBM Corp., Armonk, NY, USA) and jamovi 2.3.5. Categorical variables were summarized as frequencies and percentages, and the principal scale scores were reported as means ± standard deviations and medians (minimum–maximum). Normality was assessed using the Shapiro–Wilk test and skewness and kurtosis coefficients, with values between −1 and +1 considered indicative of an approximately normal distribution [36]. Depending on distributional characteristics, two-group comparisons were performed using Student’s t-test or the Mann–Whitney U test, and comparisons involving more than two groups were performed using one-way ANOVA or the Kruskal–Wallis test. Post hoc comparisons used Tukey’s test when homogeneity of variances was met and the Games–Howell test otherwise; pairwise comparisons following the Kruskal–Wallis test used Bonferroni-adjusted Mann–Whitney U tests. The demographic and clinical comparisons were treated as exploratory, with no global adjustment for multiple testing across the full set of comparisons. Spearman rank correlations were used to assess the associations of PITI scores with NRS and PTDS scores. Pearson correlation was used to assess the association between NRS and PTDS scores, which were treated as approximately continuous based on their measurement and distributional characteristics. Correlation magnitudes were interpreted according to Cohen’s conventions, with absolute coefficients of approximately 0.10, 0.30, and 0.50 representing small, moderate, and large associations, respectively [37]. A secondary exploratory path analysis was conducted using jamovi 2.3.5 to estimate total, direct, and indirect statistical associations among preoperative PITI scores, postoperative NRS scores, and postoperative PTDS scores. The statistical significance of the indirect association was assessed. Because PITI was assessed preoperatively, whereas NRS and PTDS were assessed concurrently at the sixth postoperative hour, the indirect association was interpreted as exploratory and not as evidence of temporal or causal mediation. There were no missing data for the variables included in the analyses; therefore, no imputation was required. Statistical significance was set at *p* < 0.05 for all analyses.

### 2.7. Ethical Considerations of the Study

Ethical approval was obtained from the Ethics Committee for Non-Interventional Clinical Research at Burdur Mehmet Akif Ersoy University (Meeting Date: 16 April 2025, Meeting No: 2025/3, Decision No: GO 2025/1406). Preliminary hospital approval was obtained from the institution where the study was conducted, and the necessary official permission was obtained from the Provincial Health Directorate (No: 49810142/806.02.02). In addition, the relevant permissions were obtained for the scales used in the study. The principles of the Declaration of Helsinki were adhered to throughout the research process; informed consent was obtained from participants based on the principle of voluntariness.

## 3. Results

The frequency distributions of the demographic characteristics of the patients included in the study are presented in Table 1. It was determined that 73.1% of the patients participating in the study were female, that the highest proportion in terms of educational status was among elementary school graduates (44.6%), and that 59.2% were classified as ASA Class II. The mean age of the patients was 52.21 ± 14.80 years; the mean preoperative fasting duration for solid foods was 12.25 ± 2.20 h, and the mean preoperative fasting duration for liquids was 11.31 ± 2.07 h (Table 1).

The mean total PITI score was 18.78 ± 11.43, while the mean NRS and PTDS scores were 5.23 ± 2.08 and 5.58 ± 2.72, respectively (Table 2).

No statistically significant differences in PITI total scores, postoperative pain intensity, or thirst discomfort were observed according to educational status, presence of chronic disease, tobacco or alcohol use, or previous surgical experience (*p* > 0.05; Table 3). Female patients had a higher mean PITI score than male patients (20.02 ± 11.05 vs. 15.43 ± 11.92), corresponding to an observed mean difference of 4.59 points (Mann–Whitney U test, *p* = 0.018). Because this comparison formed part of the exploratory subgroup analyses presented in Table 3, the statistical significance of the difference should not, by itself, be interpreted as evidence of clinical importance. Overall differences in PITI total and subscale scores were statistically significant across ASA categories (*p* < 0.05; Table 3). However, the ASA III subgroup included only five participants; these exploratory findings should therefore be interpreted cautiously and do not support firm conclusions about differences involving this subgroup.

**Table 3:** Comparison of Mean Scores for Intrusive Thoughts, Pain Intensity, and Thirst Discomfort According to Patient Characteristics.

	Mean ± SD Median (Min–Max)				
	Intrusive Thoughts	Anxiety About Loss of Control	Anxiety Related to the Post-Procedure Period	Pain Intensity	Thirst Discomfort
**Gender**					
Female	20.02 ± 11.05 17.00 (1.00–45.00)	8.61 ± 5.65 7.00 (0.00–23.00)	11.41 ± 6.17 10.00 (0.00–25.00)	5.41 ± 2.01 5.00 (0.00–10.00)	5.67 ± 2.66 5.00 (0.00–12.00)
Male	15.43 ± 11.92 11.00 (0.00–48.00)	6.06 ± 5.97 4.00 (0.00–25.00)	9.37 ± 6.63 8.00 (0.00–23.00)	4.74 ± 2.21 5.00 (0.00–9.00)	5.31 ± 2.90 5.00 (1.00–12.00)
***p*** **value**	***0.018 ^b^***	***0.004 ^b^***	0.099 ^b^	0.107 ^a^	0.411 ^a^
**Educational status**					
Primary school	17.43 ± 10.26 15.00 (1.00–45.00)	6.90 ± 4.85 6.00 (0.00–22.00)	10.53 ± 5.99 9.00 (0.00–24.00)	5.26 ± 1.86 5.00 (0.00–10.00)	5.28 ± 2.68 5.00 (1.00–11.00)
Middle school	18.64 ± 13.32 13.00 (3.00–48.00)	7.71 ± 7.67 5.00 (0.00–25.00)	10.93 ± 6.04 9.50 (2.00–23.00)	5.71 ± 2.02 5.00 (3.00–9.00)	6.21 ± 3.81 6.00 (0.00–12.00)
High school	20.10 ± 11.17 19.00 (0.00–44.00)	8.67 ± 5.25 7.00 (0.00–23.00)	11.43 ± 6.65 10.50 (0.00–25.00)	5.27 ± 2.57 5.00 (0.00–10.00)	5.83 ± 2.61 6.00 (1.00–10.00)
University	20.25 ± 13.20 21.00 (0.00–45.00)	9.36 ± 7.02 9.00 (0.00–22.00)	10.89 ± 7.09 10.00 (0.00–23.00)	4.89 ± 2.02 5.00 (1.00–8.00)	5.61 ± 2.30 5.00 (2.00–11.00)
***p*** **value**	0.703 ^d^	0.256 ^d^	0.947 ^d^	0.854 ^c^	0.677 ^c^
**Chronic disease**					
No	19.92 ± 12.24 17.00 (0.00–45.00)	8.69 ± 6.37 7.00 (0.00–23.00)	11.23 ± 6.68 10.00 (0.00–25.00)	5.10 ± 2.13 5.00 (0.00–10.00)	5.48 ± 2.55 5.00 (0.00–12.00)
Yes	17.75 ± 10.62 15.50 (1.00–48.00)	7.22 ± 5.23 6.00 (0.00–25.00)	10.53 ± 6.03 9.00 (0.00–23.00)	5.35 ± 2.04 5.00 (0.00–10.00)	5.66 ± 2.87 5.00 (1.00–12.00)
***p*** **value**	0.395 ^b^	0.258 ^b^	0.550 ^b^	0.404 ^a^	0.815 ^a^
**Tobacco use**					
Yes	18.31 ± 10.77 16.00 (0.00–44.00)	7.93 ± 5.61 7.00 (0.00–21.00)	10.38 ± 5.70 10.00 (0.00–23.00)	5.52 ± 2.21 5.00 (0.00–9.00)	5.21 ± 2.83 5.00 (0.00–12.00)
No	18.92 ± 11.66 16.00 (1.00–48.00)	7.92 ± 5.91 7.00 (0.00–25.00)	11.00 ± 6.52 10.00 (0.00–25.00)	5.15 ± 2.05 5.00 (0.00–10.00)	5.68 ± 2.69 5.00 (1.00–12.00)
***p*** **value**	0.889 ^b^	0.940 ^b^	0.823 ^b^	0.317 ^a^	0.408 ^a^
**Alcohol use**					
Yes	21.75 ± 12.42 23.50 (7.00–33.00)	8.00 ± 4.76 8.00 (3.00–13.00)	13.75 ± 7.93 14.50 (4.00–22.00)	6.25 ± 1.71 6.50 (4.00–8.00)	6.00 ± 3.56 6.50 (2.00–9.00)
No	18.84 ± 11.51 16.00 (0.00–48.00)	7.98 ± 5.86 7.00 (0.00–25.00)	10.85 ± 6.33 10.00 (0.00–25.00)	5.21 ± 2.03 5.00 (0.00–10.00)	5.62 ± 2.70 5.00 (0.00–12.00)
Occasionally	16.78 ± 10.87 16.00 (0.00–30.00)	7.11 ± 6.35 5.00 (0.00–18.00)	9.67 ± 6.10 10.00 (0.00–20.00)	5.11 ± 2.89 5.00 (2.00–10.00)	4.78 ± 2.73 4.00 (2.00–10.00)
***p*** **value**	0.798 ^d^	0.805 ^d^	0.675 ^d^	0.533 ^c^	0.589 ^c^
**Surgical experience**					
Yes	18.72 ± 11.74 16.00 (0.00–48.00)	7.74 ± 5.84 6.00 (0.00–25.00)	10.98 ± 6.59 10.00 (0.00–25.00)	5.29 ± 1.98 5.00 (0.00–10.00)	5.52 ± 2.69 5.00 (0.00–12.00)
No	18.95 ± 10.80 16.50 (1.00–44.00)	8.37 ± 5.83 7.00 (0.00–23.00)	10.58 ± 5.74; 10.00 (0.00–21.00)	5.08 ± 2.34 5.00 (0.00–10.00)	5.71 ± 2.82 5.00 (1.00–12.00)
***p*** **value**	0.788 ^b^	0.438 ^b^	0.825 ^b^	0.570 ^a^	0.777 ^a^
**ASA classification**					
ASA I (n: 48)	21.08 ± 12.43 20.00 (0.00–45.00)	9.29 ± 6.57 7.00 (0.00–23.00)	11.79 ± 6.71 10.00 (0.00–25.00)	5.38 ± 1.99 5.00 (2.00–10.00)	5.67 ± 2.70 5.50 (0.00–12.00)
ASA II (n: 77)	18.05 ± 10.62 16.00 (1.00–48.00)	7.35 ± 5.27 6.00 (0.00–25.00)	10.70 ± 6.03 10.00 (0.00–23.00)	5.13 ± 2.15 5.00 (0.00–10.00)	5.60 ± 2.77 5.00 (1.00–12.00)
ASA III (n: 5)	8.00 ± 5.83 7.00 (2.00–15.00)	3.60 ± 2.70 2.00 (1.00–7.00)	4.40 ± 3.29 5.00 (1.00–8.00)	5.40 ± 2.30 6.00 (3.00–8.00)	4.40 ± 2.19 4.00 (2.00–8.00)
***p*** **value**	***0.034 ^d^***	***0.045 ^d^***	***0.042 ^d^***	0.803 ^c^	0.611 ^c^

SD: Standard deviation; ^a^: Independent-samples *t*-test; ^b^: Mann–Whitney *U* test; ^c^: ANOVA; ^d^: Kruskal–Wallis test. No global adjustment for multiple testing was applied across all comparisons in this table; therefore, statistically significant findings should be interpreted as exploratory. Bold and italic values indicate statistically significant results (*p* < 0.05).

Moderate positive and statistically significant correlations were found between the total PITI score and pain intensity (r = 0.320) and thirst discomfort (r = 0.303) (*p* < 0.001; Table 4). In addition, a weak positive and statistically significant correlation was found between pain intensity and thirst discomfort (r = 0.191; *p* < 0.05; Table 4).

In the secondary exploratory path analysis, the indirect statistical effect estimated in the specified model involving postoperative pain intensity, preoperative PITI scores, and thirst discomfort was statistically significant (indirect effect = 0.105, SE = 0.0448, 95% CI [0.0169, 0.192], *p* = 0.019). Neither the direct effect (B = 0.104, SE = 0.112, 95% CI [−0.115, 0.323], *p* = 0.351) nor the total effect (B = 0.209, SE = 0.113, 95% CI [−0.013, 0.430], *p* = 0.065) reached statistical significance. The model coefficients indicated statistically significant associations between pain intensity and PITI scores (B = 1.4264, SE = 0.4650, 95% CI [0.5151, 2.338], *p* = 0.002) and between PITI scores and thirst discomfort (B = 0.0734, SE = 0.0203, 95% CI [0.0335, 0.113], *p* < 0.001). All reported path coefficients and effect estimates are unstandardized. Because PITI was assessed preoperatively, whereas pain intensity and thirst discomfort were assessed concurrently in the postoperative period, the specified path was interpreted as an exploratory statistical ordering rather than as evidence of a temporal sequence or causal mediation (Table 5).

## 4. Discussion

The mean PITI total score was 18.78 ± 11.43. Previous studies reported mean scores of 28.92 ± 14.09 [32] and 9.11 ± 10.20 [38]. These comparisons are descriptive; differences in sample characteristics, surgical procedures, and previous surgical experiences may account for some of the variation. Female patients had higher total PITI scores than male patients, consistent with previous studies reporting higher scores among women [23,32,39]. In contrast, another study found no statistically significant difference in PITI scores by sex [40]. The unequal group sizes in the present study should be considered when interpreting this comparison.

No statistically significant differences in PITI total or subscale scores were observed according to educational status, chronic disease, tobacco or alcohol use, or previous surgical experience. Kuyumcu (2022) similarly reported no significant differences according to educational status, tobacco or alcohol use, or surgical experience [40]. A study of patients undergoing ureteroscopy also found no significant associations between PITI total scores and several sociodemographic and clinical characteristics [39]. These results do not establish that such characteristics are unrelated to preoperative intrusive thoughts. Hinuma et al. (2025) discussed cognitive and emotional processes, including negative cognitive appraisal, attempts to control thoughts, and stress responses, in relation to intrusive thoughts [41]. However, these processes were not assessed separately in the present study, and their contribution cannot be compared with that of the demographic and clinical characteristics examined.

The mean postoperative NRS score was 5.23 ± 2.08, numerically higher than the mean pain score of 4.43 ± 2.12 reported by İşeri et al. (2025) in general surgery and neurosurgery patients [24]. This descriptive difference may reflect variation in surgical procedures, patient characteristics, and assessment conditions. Although previous literature has reported greater postoperative pain among women [42], no statistically significant sex difference in pain intensity was observed in the present study. Women accounted for 73.1% of the sample, and the male subgroup included only 35 participants, which may have limited the ability to detect a difference.

The mean PTDS total score was 5.58 ± 2.72. A study of general surgery patients reported a mean thirst score of 5.66 ± 3.18 at the second postoperative hour [35], while another study using an NRS (0–10) reported a mean score of 4.39 ± 2.976 [43]. Differences in measurement instruments and assessment timing limit direct comparison of these scores. Sample characteristics and perioperative care practices may also contribute to the variation. All participants in the present study received 100 mg of intravenous tramadol as part of the standardized postoperative analgesic protocol. Dry mouth is a recognized adverse effect of tramadol [44] and may have contributed to the reported thirst discomfort, although this contribution was not assessed separately.

Findings on the relationship between sex and postoperative thirst also vary. Some studies reported no statistically significant differences by sex [45,46], whereas others identified a significant association [47]. A meta-analysis identified sex as a potential risk factor but did not establish which sex experiences greater thirst [11]. In the present study, no statistically significant sex difference in postoperative thirst discomfort was observed. As with pain intensity, the smaller male subgroup warrants caution in interpreting this result. Differences in how patients perceive and report physical sensations may also contribute to variation across studies.

PTDS total scores did not differ significantly according to chronic disease, tobacco or alcohol use, previous surgical experience, or ASA classification. Studies of abdominal surgery patients likewise reported no significant differences in postoperative thirst according to smoking status, alcohol use, or ASA classification [24,47]. Belete et al. (2022) did not identify ASA classification or a history of comorbidity as significant factors associated with the presence of postoperative thirst in the final multivariable analysis [43]. Other factors discussed in the literature include perioperative fluid restriction, anesthesia and surgical characteristics [11,43], dry mouth, and individual symptom perception [48,49]. The absence of significant group differences in the present study does not establish the relative importance of these factors.

A moderate positive correlation was observed between preoperative PITI total scores and postoperative pain intensity. Patients with higher PITI scores tended to report greater pain intensity. Associations between psychological characteristics and pain experiences have also been reported in previous research [39,50,51,52]. Gülşen et al. (2026) found that higher intrusive thought scores were associated with greater fear of pain [39]. Franqueiro et al. (2024) reported associations of preoperative anxiety and depression with greater postoperative pain [50]. Alptekin et al. (2025) described relationships among preoperative stress and anxiety, surgical fears, and reduced comfort [51], while Bekele et al. (2024) identified preoperative anxiety as a factor associated with acute postoperative pain [52]. These studies address related but distinct constructs. The Turkish PITI assesses surgery-related anxious cognitions concerning loss of control and the post-procedure period, which overlap with preoperative anxiety but should not be equated with generalized anxiety, depression, or intrusive-thought phenomena associated with psychiatric disorders.

Preoperative PITI scores were also moderately and positively correlated with postoperative thirst discomfort. Anxiety and stress have been associated with dry mouth [53]. Cognitive intrusion of pain has been associated with higher pain scores, greater fear of surgery, and lower comfort [54]. However, the psychological characteristics examined in these studies differ from the construct assessed by the PITI, and these findings do not directly explain the association between preoperative intrusive thoughts and postoperative thirst discomfort. The potential role of dry mouth in this association could not be evaluated because it was not measured in the present study.

The correlation between postoperative pain intensity and thirst discomfort was weak and positive. Research examining this relationship remains limited [43,46]. A cross-sectional study of abdominal surgery patients identified postoperative pain as a predictor of thirst within a regression model that explained 64% of the variance in thirst [46]. This percentage describes the overall model and is not directly comparable with the bivariate correlation reported here. Shared perioperative conditions may contribute to the association between pain and thirst discomfort, but this possibility was not directly tested. Their concurrent assessment also prevents conclusions about whether either symptom influences the other.

These associations can be discussed within a biopsychosocial framework alongside literature on fluid balance, perioperative fluid loss, and fasting duration [43], thirst perception [55], and emotional distress [13]. Associations of preoperative intrusive thoughts and sleep quality with fear of pain have also been reported [23]. However, using this framework does not establish a psychological mechanism or demonstrate that PITI scores explain postoperative symptoms beyond physiological and perioperative factors. Potentially relevant perioperative covariates were not included in the analyses.

The secondary exploratory path analysis identified a statistically significant indirect association within the specified model, whereas neither the direct nor the total effect reached statistical significance. PITI was assessed preoperatively, while pain intensity and thirst discomfort were assessed concurrently at the sixth postoperative hour. This measurement timing does not support interpreting the specified pain → intrusive thoughts → thirst discomfort ordering as a temporal process. The indirect association should therefore be regarded as an exploratory, model-dependent finding rather than evidence of causal mediation. Further research with theoretically justified measurement schedules and assessment of relevant perioperative factors is needed to clarify these relationships.

### Limitations of the Study

The study was conducted at a single center among patients undergoing the same surgical procedure performed by a single surgeon, limiting the generalizability of the findings to other surgical populations and clinical settings. The unequal sex distribution and the small number of participants in the ASA III subgroup may also have reduced the precision of subgroup comparisons. The use of self-report measures to assess pain intensity, thirst discomfort, and intrusive thoughts may have made the measurements susceptible to individual differences in perception and reporting. Although postoperative analgesia and ward fluid therapy were standardized, the analyses did not adjust for patient-level variation in total perioperative opioid exposure, intraoperative fluid loss, or the duration of surgery and anesthesia. Other potentially relevant variables, including postoperative nausea and vomiting, dry mouth, and hydration status, were also not included in the analyses. Residual confounding cannot be excluded and may have contributed to or exaggerated the observed association between preoperative intrusive thoughts and postoperative thirst discomfort.

PITI was assessed preoperatively, whereas pain intensity and thirst discomfort were assessed concurrently at the sixth postoperative hour. This measurement timing does not permit the pain → intrusive thoughts → thirst discomfort ordering specified in the path model to be interpreted as a temporal process. The indirect association should therefore be regarded as an exploratory statistical finding dependent on the specified model rather than as evidence of causal mediation. Assessing pain intensity and thirst discomfort only at the sixth postoperative hour limited the examination of changes over time and the generalizability of the findings to other stages of postoperative recovery.

The absence of a global adjustment for multiple testing across the demographic and clinical comparisons increases the possibility of chance findings. In addition, no a priori power analysis was performed specifically for the indirect effect examined in the exploratory path analysis. The lower bound of the confidence interval for the indirect effect was close to zero (95% CI [0.0169, 0.192]), warranting caution in interpreting this exploratory finding. The findings should be evaluated in larger, multicenter studies with repeated measurements scheduled according to a theoretically justified temporal ordering of the variables.

Patients with a history of psychiatric or neurological disorders identified through patient reports and medical record review were excluded from the study. This exclusion may have reduced the variability of preoperative psychological responses and limited the representativeness of the sample. Although patient reports were checked against medical records, no structured diagnostic assessment was performed, and misclassification cannot be excluded. These considerations limit the generalizability of the findings to surgical patients with psychiatric or neurological comorbidities.

## 5. Conclusions

Preoperative intrusive thoughts were moderately and positively associated with postoperative pain intensity and thirst discomfort, while a weak positive association was observed between the two postoperative symptoms. These findings suggest that preoperative cognitive and psychological characteristics may warrant consideration when evaluating postoperative symptoms within a biopsychosocial framework. The indirect association observed in the path analysis remains exploratory. Longitudinal research is needed to clarify the temporal relationships among these variables and the processes underlying their associations.

## Figures and Tables

**Table 1:** Patient Characteristics and Related Findings.

	Number (n)	Percentage (%)
**Gender**		
Female	95	73.1
Male	35	26.9
**Educational status**		
Primary school	58	44.6
Middle school	14	10.8
High school	30	23.1
University	28	21.5
**Chronic disease**		
No	62	47.7
Yes	68	52.3
**Tobacco use**		
Yes	29	22.3
No	101	77.7
**Alcohol use**		
Yes	4	3.1
No	117	90.0
Occasionally	9	6.9
**Surgical experience**		
Yes	92	70.8
No	38	29.2
**ASA classification**		
ASA I	48	36.9
ASA II	77	59.2
ASA III	5	3.8
	**Mean ± SD**	**Min–Max**
**Age**	52.21 ± 14.80	18–79
Fasting Duration _(Solid)_	12.25 ± 2.20	8–18
Fasting Duration _(Liquids)_	11.31 ± 2.07	8–20

SD: Standard deviation; ASA: American Society of Anesthesiologists physical status classification.

**Table 2:** Descriptive Statistics for Preoperative Intrusive Thoughts, Postoperative Pain Intensity, and Thirst Discomfort.

	Mean ± SD	Median (Min–Max)
**Intrusive Thoughts**	18.78 ± 11.43	16.00 (0.00–48.00)
Anxiety about loss of control	7.92 ± 5.82	7.00 (0.00–25.00)
Anxiety related to the post-procedure period	10.86 ± 6.33	10.00 (0.00–25.00)
**Pain Intensity**	5.23 ± 2.08	5.00 (0.00–10.00)
**Thirst Discomfort**	5.58 ± 2.72	5.00 (0.00–12.00)

SD: Standard deviation. For descriptive completeness, the principal scale scores are presented as both mean ± standard deviation and median (min–max). Inferential analyses were selected according to the distributional characteristics of the variables.

**Table 4:** Correlations Among Preoperative Intrusive Thoughts, Postoperative Pain Intensity, and Thirst Discomfort.

Variable Pair	r	*p*-Value	Correlation Method
PITI score—Pain intensity	0.320	<0.001	Spearman
PITI score—Thirst discomfort	0.303	<0.001	Spearman
Pain intensity—Thirst discomfort	0.191	0.029	Pearson

**Table 5:** Direct and Indirect Statistical Effects Estimated in the Secondary Exploratory Path Model Involving Postoperative Pain Intensity, Preoperative PITI Scores, and Thirst Discomfort.

Model Pathway (Indirect Effect)	Estimate	Standard Error (SE)	95% Confidence Interval (CI)		*p*-Value
			Lower	Upper	
Pain Intensity → Intrusive Thoughts → Thirst Discomfort	0.105	0.045	0.017	0.192	0.019
Direct Effect	0.104	0.112	−0.115	0.323	0.351
Total effect	0.209	0.113	−0.013	0.430	0.065

Note: Estimates are unstandardized. The ordering of variables reflects the statistical specification of the exploratory path model and should not be interpreted as representing temporal or causal mediation.

## Data Availability

Data are not publicly available due to ethical and privacy considerations. De-identified data may be made available by the corresponding author, H.Ö. (htcozsoy15@gmail.com), upon reasonable request, provided that such sharing is consistent with the participants’ informed consent and applicable ethical requirements.

## Abbreviations

The following abbreviations are used in this manuscript:

ASA	American Society of Anesthesiologists
BMI	Body Mass Index
NRS	Numerical Rating Scale
PITI	Preoperative Intrusive Thoughts Inventory
PTDS	Perioperative Thirst Discomfort Scale
